# Social comparison threat and coping: affective, cognitive and behavioral outcomes in response to false performance-related feedback

**DOI:** 10.1186/s40359-026-05536-9

**Published:** 2026-09-09

**Authors:** Thomas Meyer, Ann-Kathrin Zenses, Nexhmedin Morina

**Affiliations:** Institute of Psychology, University of Münster, Münster, Germany

**Keywords:** Social comparison, Affect, Cognition, Behavior, False performance feedback, GComp

## Abstract

**Background:**

Social comparisons shape our behavior. The general comparative processing model (gComp) suggests that evaluating one’s attributes against salient upward social standards elicits motivational appraisal ranging from threat to challenge, each typically associated with corresponding pessimistic or optimistic coping responses. This, in turn, manifests in specific affective, cognitive, and behavioral responses. To our knowledge, this is the first study to provide an initial experimental test of gComp assumptions.

**Methods:**

Participants completed a 2-back task framed as indicative of academic performance. They were randomly assigned to high-threat (*n* = 67) or low-threat (*n* = 62) conditions, receiving false social comparison feedback on their performance relative to that of previous participants. Outcome measures included affect, cognitive responses (i.e., rededication, distraction, reconstrual, commitment), and a behavioral choice (i.e., task repetition).

**Results:**

Participants in the high-threat condition reported significantly higher increases in negative affect (*p* < .001), greater rededication (i.e., downplaying the task’s importance; *p* = .011), and lower commitment (*p* < .001) compared to those in the low-threat condition. However, no significant differences were found for the behavioral outcome measure (*p* = .149).

**Conclusions:**

The findings underscore the potential of a process-based approach to social comparison to advance our understanding of how comparative feedback shapes affective, cognitive, and behavioral responses.

## Background

Social comparison, defined as the evaluation of self-attributes relative to others [1], profoundly influences emotions, cognitions, and behaviors. Research suggests that individuals generally prefer upward (i.e., against superior others) over downward (i.e., against inferior others) comparisons, with threat amplifying this tendency [2]. Frequent upward social comparisons typically result in significant declines in perceived ability, whereas frequent downward comparisons boost self-appraisals. Similarly, upward comparisons frequently elicit negative emotions [3, 4]. With respect to behavioral reactions, upward comparisons can have dual behavioral effects. They may motivate individuals toward self-improvement when the comparison target’s success seems attainable, leading to increased effort towards the desired end state. Conversely, if the success appears unattainable, such comparisons can demotivate [5, 6].

A process-based account of social comparison is important for understanding why unfavorable comparisons sometimes undermine affect, motivation, and performance, whereas in other cases they may motivate self-improvement. Wood [7] defined social comparison as a process involving thinking about social information in relation to the self, which, in turn, can elicit cognitive, affective, and behavioral responses. Experimental research has distinguished two broad approaches to studying social comparison [7], a taxonomy that continues to guide contemporary research [2, 8, 9]. Selection studies examine the conditions under which individuals choose particular comparison standards, whereas reaction studies investigate the consequences of exposure to social comparison information. In the latter approach, social comparison information constitutes the independent variable, and researchers examine how individuals respond when they are informed that others perform better or worse on a self-relevant dimension. Accordingly, this experimental approach induces social comparison through comparative performance feedback by explicitly relating participants’ performance to that of others. Performance represents one of the most frequently investigated comparison dimensions, with studies commonly manipulating whether participants are informed that they performed better or worse than other individuals and subsequently assessing their affective, cognitive, or behavioral responses [2].

Relatedly, a large-scale meta-analysis on social comparison by Gerber et al. [2] reported that controlled experimental studies involving manipulated exposure to comparison standards produce significant effects on perceived ability and performance satisfaction. However, these experimental studies did not, on average, produce any significant or consistent effects on affect or behavior. This suggests that the effects of comparative feedback on affective and behavioral outcomes may depend on intervening appraisal and coping processes that are not captured by exposure to comparison information alone.

Building on this well-established reaction paradigm, the present study experimentally manipulated comparative performance feedback and examined selected affective, cognitive and behavioral responses. To specify the process through which exposure to social comparisons influences affect, cognition, and behavior, Morina [6] proposed a comprehensive framework encompassing social and related comparison types. The general comparative processing model of self-perception (gComp) builds on Wood’s conceptualization of the social comparison process while further proposing that the outcome of a comparison between a target (e.g., a student’s own academic performance) and standard (e.g., another student´s academic performance) is evaluated against its motivational meaning, which may then engender different affective, cognitive and behavioral responses.

Within this process, gComp emphasizes the role of cognitive processing in shaping behavioral outcomes. It suggests first that appraisals of the motivational meaning of the comparison outcome (i.e., whether one is better off, similar, or worse off than the comparison standard) yield four potential valuation outcomes: irrelevant, threat, challenge, and consonant. Valuation outcomes deemed as irrelevant (e.g., “*Jon is a better piano player*,* but I don’t care much about playing piano*”) or consonant (e.g., “*Jon is a good student*,* and we received the same grade on this exam*,* so I’m on the right track*”) are less likely to prompt significant behavioral consequences. In contrast, challenge and threat refer to distinct evaluations of a comparison outcome. A comparison outcome may challenge personally important motives while still being accompanied by optimism that the desired end state can be achieved in the future. Conversely, a comparison outcome may threaten personal motives and be accompanied by pessimism that the desired end state cannot be achieved. Thus, threat refers to the evaluation that one’s current standing relative to the comparison standard is unlikely to be overcome, whereas challenge refers to the evaluation that the discrepancy remains attainable despite the unfavorable comparison. The present study focuses on feedback conditions intended to elicit the latter two valuation outcomes, threat and challenge.

gComp predicts that a comparison outcome valuated as threatening and beyond one’s coping capacity is likely to elicit heightened negative affect. As a result, cognitive orientation may be focused on *distraction* from the comparison outcome and its meaning, *rededication* (i.e., deeming the comparison outcome as less important) or *reconstrual* (i.e., attributing the comparison outcome to other, usually external, factors). Distraction and rededication, in turn, may foster behavioral quiescence in response to the comparison: a reduction in effort directed toward achieving the desired end state. Reconstrual, in contrast, is unlikely to produce direct behavioral effects [6]. On the other hand, gComp proposes that when a comparison outcome is valuated as challenging but within one’s coping capacity, it is likely to elicit emotional excitement. The corresponding cognitive orientation then centers on the comparer’s *commitment* towards achieving the desired end state, which should ultimately result in behavioral assimilation.

Importantly, the proposed link between valuation pathways and affect, cognitive orientation, and behavior has not been experimentally tested yet. With this in mind, we manipulated performance-related comparison feedback intended to elicit high- vs. low-threat valuations to investigate its effect on affective, cognitive and behavioral responses. In accordance with gComp, the high-threat condition was intended to instantiate the proposed threat pathway by combining an academically self-relevant task framing with unfavorable relative performance feedback. In contrast, the low-threat condition was intended to foster a challenge-like valuation by providing more favorable (i.e., less threatening) relative performance feedback while still leaving room for future improvement. Specifically, participants performed a 2-back task, which was introduced to participants as a predictor of academic performance. Participants were randomly assigned to either a high-threat or low-threat condition, in which they received false feedback regarding their performance relative to that of previous participants. The main outcome variables were affect, assessed before and after the task with the Positive and Negative Affect Schedule (PANAS [10]; German version [11]), cognitive responses (assessed with self-rating items specifically developed for this study) and overt behavior by measuring participants’ choice to repeat the task.

Thus, we tested selected assumptions of gComp regarding affective, cognitive, and immediate behavioral responses following performance-related comparative feedback. We hypothesized that participants in the high-threat group would report a greater increase in negative affect and a greater decrease in positive affect than those in the low-threat group, following the false performance-related social comparison feedback. This pattern would replicate prior studies that have used similar feedback to induce affective responses [12]. Regarding cognitive response, we predicted that participants in the high-threat group would report greater reconstrual, rededication and distraction, but lower commitment than the low-threat group. Concerning the immediate behavioral response, we expected participants in the low-threat group to be more likely to repeat the task than those in the high-threat group. Overall, a pattern of findings consistent with these predictions would provide initial support for gComp’s assumptions concerning affective, cognitive, and immediate behavioral responses following performance-related comparative feedback. Conversely, deviations from these predictions would indicate that these assumptions require refinement or that additional moderating processes should be incorporated into the model.

## Method

### Openness and transparency

This study was not preregistered. It represents an initial effort to establish a valid paradigm and elucidate the different components of the comparison process triggered by false performance-related social comparison feedback, paving the way for more severe tests of theory in the future. We report considerations to determine the sample size, data exclusions from analyses, and manipulations relevant to this study. The hypotheses and main analytic strategy followed the theoretical rationale described in the manuscript; however, because they were not time-stamped in a preregistration, they should be interpreted accordingly. The suspicion-coding analyses were added during revision in response to reviewer concerns and should be considered additional robustness/descriptive analyses. The anonymized data, R analysis scripts [13] that allow reproduction of the present results, as well as the experimental instructions and their English translations, are available on the Open Science Framework (OSF) at the following link: https://doi.org/10.17605/OSF.IO/QKWRN.

### Participants

One hundred forty-three participants (age range: 18–39 years, *M* = 24.17, *SD* = 3.73) met the eligibility requirements and completed the study. Of these, 14 participants were removed due to poor performance on the 2-back task (for details, see Data Analyses and Reduction), yielding a final sample of *N* = 129 (see Table 1). Exclusion criteria were: younger than 18 years, insufficient proficiency in the German language, current depression (defined as a sum score of $\:\ge\:$10 on the Patient Health Questionnaire depression module [PHQ-9 [14], German version [15] ], or endorsement of its suicidality item) or current treatment of depression.

Participants were recruited from the student population of the University of Münster via university social media channels and newsletters. As a cover story, participants were told that the study aimed to examine individual differences in academic performance to better understand student achievement at the University of Münster. They were informed that the study involved completing a series of questionnaires and a concentration task. The term “concentration task” was used as a participant-facing description of the *n*-back task, and participants were subsequently debriefed and informed that the task was not, in fact, a diagnostic test. Participation was reimbursed with partial course credit or four Euros.

The planned sample size was determined based on our goal to detect moderate effects on affect and cognitive orientation, comparable to those often seen in false-feedback studies (e.g., *d* = 0.83; 12). Conservatively assuming a moderate effect size of *d* = 0.50, a sample of *N* = 128 was deemed sufficient to achieve 80% power with a two-sided alpha set at 0.05 [16]. Our final sample size of *N* = 129 met this criterion. A sensitivity analysis based on the final group sizes (low-threat: *n* = 62, high-threat: *n* = 67) indicated that the study had 80% power to detect effects of *d* = 0.50 or larger for two-sided independent-samples comparisons.

### PHQ-9

Given that the study involved potentially negative evaluative feedback, participants with current clinically relevant depressive symptoms were excluded as a safety measure and to reduce the likelihood that affective responses to the manipulation were strongly influenced by current depressive symptomatology. Depressive symptoms were assessed using the depression module of the Patient Health Questionnaire (PHQ-9; 14, 15; α = 0.89), a widely used brief screening instrument measuring symptom presence and severity over the past two weeks. Responses were rated on a 4-point scale from 0 (*not at all*) to 3 (*nearly every day*), and total scores were calculated by summing all items. Scores of ≥ 10 are indicative of clinically relevant depressive symptoms and suggest the presence of depression [14].

**Table 1:** Participants’ characteristics for each condition (*N* = 129; age range: 19–39 years)

	Low-threat (*n* = 62; 46 females, 16 males)	High-threat (*n* = 67; 49 females, 18 males)				
	M (SD)	M (SD)	t	df	*p*	d
Age	24.19 (2.83)	23.85 (4.04)	0.56	118.52	0.575	0.10
PHQ-9	3.40 (2.39)	3.28 (2.17)	0.30	127	0.766	0.05

Note. PHQ-9, Patient Health Questionnaire depression module

### Apparatus and stimuli

The study was conducted online. EFS Survey (Questback Gmbh) was used to screen for the exclusion criteria. The 2-back task and the self-reports were programmed and presented using Labvanced.org [17].

### 2-Back Task

**Fig. 1:**
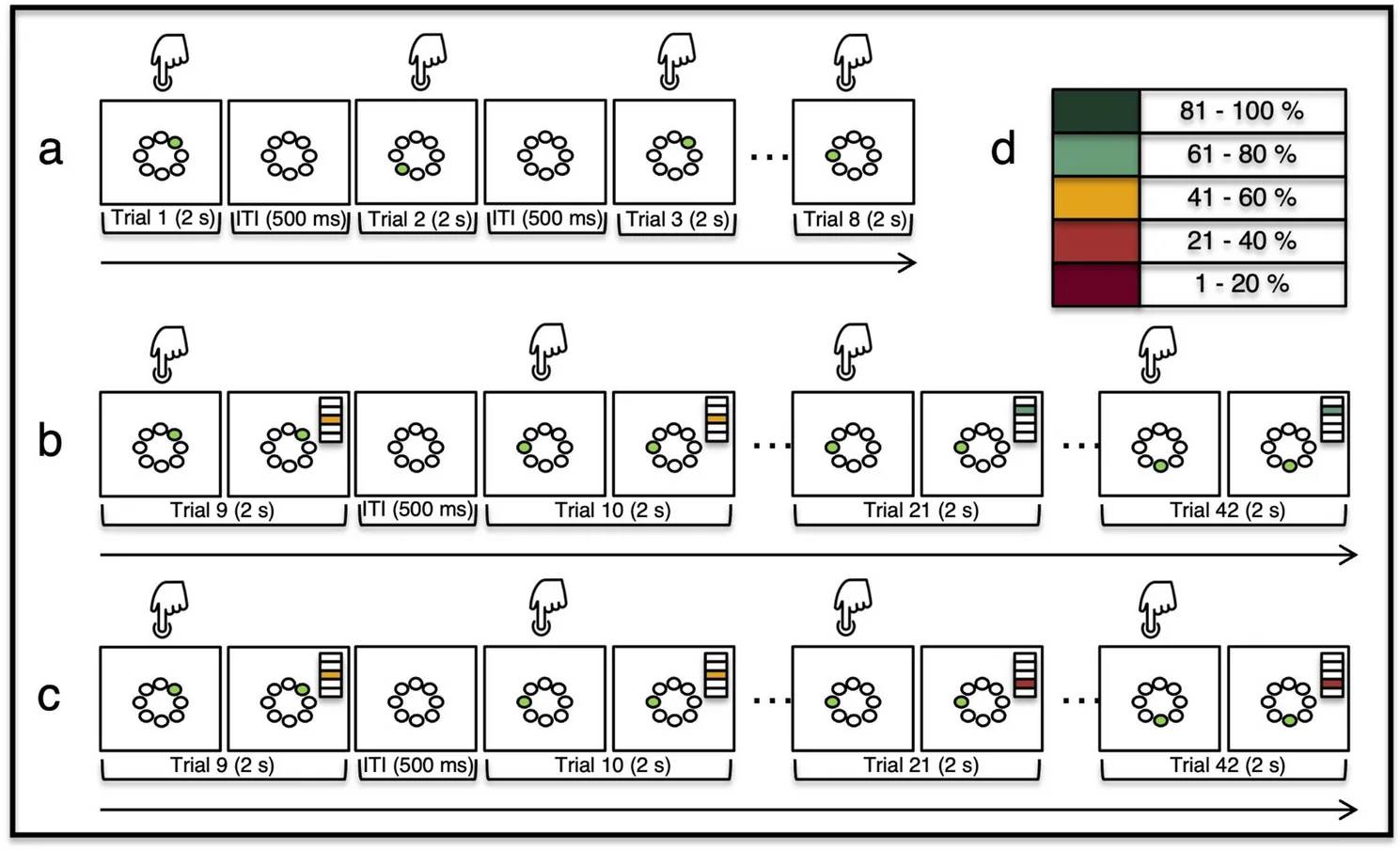
Trial Setup of the 2-Back Task. Note . The trial setup of the 2-back task is illustrated in Panels a-d. Panel a shows exemplary trials without the feedback bar. Panels b and c present exemplary trials with the feedback bar for the low-threat (Panel b) and high-threat (Panel c) groups. The feedback bar appeared after each button press in these trials. Panel d displays the feedback bar as shown to participants during the instructions. ITI = intertrial interval.

Based on an *n*-back task template provided on the platform Labvanced [17], we employed stimuli consisting of an array of eight small circles with a black outline and different fill colors (i.e., one green-filled circle and seven white-filled circles) arranged in the form of a larger circle (Fig. 1, Panel a-c), on a white screen. Images varied in the position of the green-filled circle, with eight distinct possible locations. The same images were used for the intertrial intervals (ITI), with the only difference that they included eight white-filled circles (Fig. 1, Panel a). The feedback bar consisted of five vertical segments ranging in fill-colors from dark green, light green, yellow, light red, to dark red (Fig. 1, Panel d), with higher segments reflecting better performance, from dark green at the top to dark red at the bottom. During the trials, only the color of the relevant segment was colored in its corresponding hue, while the other segments were filled with white (Fig. 1, Panel b & c). Each trial consisted of a 2000 ms image presentation, followed by an intertrial interval (ITI) of 500 ms. On each trial, participants were instructed to press the “C” key if the currently presented image matched the one shown two trials earlier (i.e., the green circle was in the same position), or the “M” key if they thought it was different.

Prior to the main task, participants completed 12 practice trials, each followed by feedback indicating whether their response was “correct” or “incorrect”. This feedback, displayed at the center of the screen, was based on participants’ actual performance in the practice trials. The main task comprised 42 trials and the sequence of images was identical for all participants. Of these, 11 were target trials and 31 were nontarget trials. During trials 1–8, the feedback bar was not presented (Fig. 1, Panel a). In the remaining trials (i.e., trials 9–42), the feedback bar appeared following each button press. During trials 9–20, all participants were shown the yellow segment of the feedback bar. From trials 21–42 onward, the feedback bar varied by experimental group: the low-threat group saw the bar increase to the light green segment (Fig. 1, Panel b), while the high-threat group saw it decrease to the light red segment (Fig. 1, Panel c).

If participants chose to perform the task again following the main task, they were administered a shorter version of the 2-back task of only 24 trials. Trials 1–4 did not include the feedback bar. Then, they received more favorable feedback than during the main task. Specifically, for participants in the low-threat group, the feedback bar changed from the light green to the dark green segment, while for those in the high-threat group, it shifted from the light red to the yellow segment.

### Outcome measures

#### Positive and negative affect

The 20-item Positive and Negative Affect Schedule (PANAS) [10], German version [11], was administered to assess positive (αs $\:\ge\:$ 0.88) and negative (αs $\:\ge\:\:$0.88) affect levels. Sum scores were computed for negative and positive affect, respectively.

#### Cognitive responses

To measure participants’ cognitive responses to social comparison information, we developed four^1^ self-report items specifically for this study (Table 2), with the German versions of the items available in the Appendix B (Table B1). All items were rated on a scale ranging from 1 (*not at all/ not essential at all*) to 7 (*very much/ very essential/ very strongly/ very important*). Higher scores on the reconstrual, distraction, and commitment items indicate greater levels of engagement in these respective cognitive strategies. In contrast, a lower score on the rededication item indicates greater rededication. **The order of the items was counterbalanced within each experimental condition**; that is, within each group, half of the participants were presented with the threat-pathway items first, while the other half received the challenge-pathway item first.

**Table 2:** Cognitive response items and their response options

Pathway	Cognitive Response	Item	Response options
Threat	Reconstrual	“To what extent did disruptive factors interfere with your performance on this task?”	1 = not at all, 7 = very strongly
	Rededication	“How important is it to you that you performed well on this task?”	1 = not essential at all, 7 = very essential
	Distraction	“To what extent do you prefer to be doing something else other than the task right now?”	1 = not at all, 7 = very much
Challenge	Commitment	“How important is it to you that you will improve your performance on similar tasks in the future?”	1 = not at all, 7 = very important

Note. Higher scores indicate greater reconstrual, distraction and commitment; lower scores indicate greater rededication

### Procedure

After providing informed consent, participants first completed an online survey, which included the PHQ-9 and the Self-Discrepancy Scale [18]. Participants who met the inclusion criteria were subsequently provided with a separate link to the online experiment. They were randomly assigned to one of two experimental conditions (i.e., high-threat or low-threat). At the beginning of the experiment, participants were reminded to perform the experiment alone and in a quiet environment with their phone being turned off. Participants first received instructions on how to perform the 2-back task. Additionally, they were informed that the concentration task measured their performance relative to other students of the University of Münster, and that previous studies have shown that performance in this task is a strong predictor of future academic success.

Following this, participants completed several practice trials of the 2-back task. They were then given more detailed explanations about the feedback system and shown an image of the feedback bar (Fig. 1, Panel d). Participants were informed that, for example, placement in the dark green segment indicates performance exceeding that of 80% of students who had previously participated in the experiment. In contrast, placement in the light red segment indicated performance exceeding that of only 20% of students. Additionally, participants were instructed to respond as quickly and accurately as possible, with their performance evaluated based on both their reaction time and error rate.

Participants were first asked to complete the PANAS, followed by the 2-back task. Afterward, they completed the PANAS again and answered the four cognitive response items (the order of presentation was random). Subsequently, they could choose to either complete a brief version of the 2-back task, lasting less than one minute, or to end the experiment. They were informed that their choice would have no impact on reimbursement or result in any negative consequences. Before debriefing, participants completed an open-ended question asking what they thought the purpose of the study was. These responses were later coded for manipulation awareness and suspicion. Finally, they were debriefed.

### Data analyses and reduction

For the affect analyses, between-group differences in the mean changes from pre-task to post-task were analyzed using mixed ANOVA. Between-group differences on the cognitive response items and 2-back performance were analyzed with independent samples Student’s *t*-tests. When Levene’s test indicated a violation of the homogeneity-of-variance assumption, Welch’s *t*-test was used. Results between the two types of *t*-tests were virtually identical. Cohen’s *d* is reported as effect size. To investigate if conditions differ in their choice to repeat the task, a Chi-square test was conducted. To quantify 2-back performance, we computed a bias-corrected discrimination index, *Pr*, using the formula ((# hits + 0.5) / (# targets + 1)) – ((# false alarms + 0.5) / (# nontargets + 1)) [19]. *Pr* reflects a response-bias-adjusted index of discrimination performance based on adjusted hit minus false alarm rates. More positive values indicate greater discrimination between target and nontarget trials. Fourteen participants from the initial sample exhibited *Pr* values $\:\le\:$ 0, indicating absent or negative discrimination, which were deemed too low for meaningful interpretation and their data were excluded from subsequent analyses. We report the analyses without applying this criterion (*N* = 143) in Appendix A (Tables A1-A5). Open-ended purpose-of-study responses were coded independently by two raters using a three-level awareness scheme (0 = no awareness, 1 = non-suspicious study-purpose awareness, 2 = manipulation awareness/suspicion). Disagreements were resolved by an independent third rater. For the primary suspicion variable, codes were dichotomized into no suspicion versus suspicion. Because exclusion based on suspicion was not specified a priori, suspicious participants were retained in the primary analyses. As a sensitivity analysis, we repeated the main inferential analyses after excluding participants classified as suspicious. All analyses were conducted in R version 4.6.1 [13] using the packages readxl, dplyr, tidyr, afex, car, emmeans, effectsize, and pwr. Alpha was set at 0.05 (two-sided) for all tests.

## Results

### Manipulation awareness and suspicion

For the three-level awareness code, exact agreement in the final analysis sample between the two raters was 61.2%; unweighted Cohen’s κ was 0.41 and quadratic-weighted Cohen’s κ was 0.64. For the primary dichotomous suspicion variable (no suspicion vs. suspicion), exact agreement was 78.3%, Cohen’s κ was 0.36. After discrepancy resolution by a third rater, 51 (39.5%) participants were coded as showing no awareness, 65 (50.4%) as showing non-suspicious study-purpose awareness, whereas 13 (10.1%) were classified as suspicious. The proportion of suspicious participants did not differ by condition, χ²(1, *N* = 129) = 0.02, *p* = .885.

### Positive and negative affect

As predicted, the high-threat group exhibited a significantly greater increase in negative affect (*M*_*Pre*_ = 13.4, *SD* = 4.0; *M*_*post*_ = 16.0, *SD* = 6.3; *M*_*Difference*_ = 2.7, *SD* = 5.0) than the low-threat group (*M*_*Pre*_ = 15.1, *SD* = 5.7; *M*_*post*_ = 14.9, *SD* = 5.3; *M*_*Difference*_ = -0.16, *SD* = 4.03), evidenced by a significant Condition × Time interaction, *F* (1,127) = 12.32, *p* < .001, η²_p_ = .088. The change in negative affect differed by 2.83 points (95% CI: 1.24, 4.43) between conditions.

At the same time, the high-threat group showed a significantly greater decrease in positive affect (*M*_*Pre*_ = 28.6, *SD* = 6.3; *M*_*post*_ = 25.8, *SD* = 6.9; *M*_*Difference*_ = -2.73, *SD* = 6.17) than the low-threat group (*M*_*Pre*_ = 29.2, *SD* = 7.0; *M*_*post*_ = 30.6, *SD* = 6.8; *M*_*Difference*_ = 1.39, *SD* = 5.15), as indicated by a significant Condition × Time interaction *F* (1,127) = 16.80, *p* < .001, η²_p_ = .117. The change in positive affect differed by -4.12 points (95% CI: -6.11, -2.13) between conditions.

### Cognitive responses

There were significant between-group differences on the cognitive responses on items for rededication and commitment. Specifically, the high-threat group reported more rededication, *t* (127) = 2.58, *p* = .011, *d* = 0.45, and less commitment, *t* (127) = 7.53, *p* < .001, *d* = 1.33, than the low-threat group (Table 3). Conditions did not significantly differ in reconstrual, *t* (127) = -1.69, *p* = .094, *d* = -0.30, and distraction, *t* (127) = -1.18, *p* = .242, *d* = -0.21.

**Table 3:** Cognitive responses for each condition (*N* = 129)

	Low-threat	High-threat				
	M (SD)	M (SD)	t	df	*p*	d
Rededication^a^	3.76 (1.59)	3.00 (1.74)	2.58	127	0.011^*^	0.45
Reconstrual	2.76 (1.55)	3.25 (1.76)	-1.69	127	0.094	-0.30
Distraction	3.65 (1.66)	4.03 (2.02)	-1.18	127	0.242	-0.21
Commitment	4.11 (1.40)	2.27 (1.38)	7.53	127	< 0.001^**^	1.33

Note. Items were rated on a scale ranging from 1 (*not at all/ not essential at all*) to 7 (*very much/ very essential/ very strongly/ very important*).^a^ Lower scores indicate higher levels of rededication.* *p* < .05, ** *p* < .001.

### Behavior

#### 2-Back Performance

During the 2-back task, the high-threat group exhibited a significantly lower discrimination performance in terms of *Pr* (*M* = 0.59, *SD* = 0.23) than the low-threat group (*M* = 0.68, *SD* = 0.22), *t* (127) = 2.15, *p* = .033, *d* = 0.38. More fine-grained follow-up analyses indicate that this performance difference was not detectable during the first 20 trials (i.e., where feedback did not yet differ between conditions; *M*_*Diff*_ = 0.01, *SE*_*Diff*_ = 0.05; *t (127)* = 0.27, *p* = .791, *d* = 0.05), but emerged in the final 22 trials (i.e., where feedback differed between conditions; *M*_*Diff*_ = 0.15, *SE*_*Diff*_ = 0.04; *t(121.90)* = 3.63, *p* < .001, *d* = 0.64).

### Willingness to Repeat the Task

Contrary to our hypothesis, the high and low-threat group did not significantly differ in their choice to repeat the 2-back task, χ ^2^ (1) = 2.09, *p* = .149 (Table 4).

**Table 4:** Number of participants repeating vs. not repeating the task by condition (*N* = 129)

Repeated task	Low-threat	High-threat	Total
	40 (64.5%)	51 (76.1%)	91
Did not repeat task	22 (35.5%)	16 (23.9%)	38
Total	62	67	

### Sensitivity Analyses Excluding Suspicious Participants

Sensitivity analyses excluding the 13 participants classified as suspicious yielded the same substantive pattern of findings. The affective and key cognitive effects remained significant, and task repetition remained non-significant. For 2-back performance, the theoretically relevant condition difference during the final trials, when feedback differed between conditions, remained significant, whereas the early-trial difference remained non-significant. Thus, the main conclusions were robust to excluding suspicious participants. Detailed sensitivity analyses are available on OSF.

## Discussion

The present study aimed to test the threat and challenge pathways proposed in gComp [6] by manipulating performance-related social comparison feedback and investigating its effect on affective, cognitive, and behavioral responses. In line with our hypotheses, we observed a greater increase in negative affect and greater decrease in positive affect for the high-threat group than for the low-threat group. Our predictions were partially supported with regard to cognitive responses. Specifically, and in line with our hypotheses, participants who received more threatening social performance feedback rated good task performance as less important, indicating greater rededication, as compared with participants in the low-threat group. Additionally, they reported lower commitment to future performance on similar tasks than participants who received less threatening feedback. Contrary to our predictions, however, no significant between-group differences were observed in either distraction (i.e., their desire to engage in other activities), or reconstrual (i.e., their tendency to attribute performance outcomes to external factors). With regard to the behavioral responses, we found no evidence of differences between the high- and low-threat groups, as their decision to repeat the task did not differ significantly between the groups. Yet, the high-threat group performed significantly worse on the 2-back task compared to the low-threat group in response to the experimental manipulation.

In general, the manipulation varied the comparison outcome in a way that was intended to elicit threat-like versus challenge-like valuations. The observed affective responses were consistent with this interpretation, as the high-threat condition showed a more negative affective pattern than the low-threat condition. This allowed us to examine experimental effects on affective responses, cognitive orientations, and immediate behavioral choice. The findings are broadly consistent with the predictions of gComp [6]. Specifically, a more threatening comparison outcome - such as that intended in the high-threat group - was associated with more pessimistic coping, as suggested by a larger increase in negative affect and poorer task performance. Previous studies have shown that false negative feedback in performance contexts, especially when framed as diagnostic of ability or intelligence, can induce heightened negative affect due to increased self-relevant threat [12, 20]. While the current paradigm employed a controlled, laboratory-based manipulation, future research may benefit from incorporating more ecologically valid designs. For instance, Schmidt, Warns [21] employed a job interview scenario in which participants received either positive or negative feedback from a fictive expert, successfully inducing changes in affective states such as valence, dominance, and self-esteem. Although their approach did not involve social comparison, it provides a compelling example of a naturalistic feedback context that could be adapted to investigate comparative threat in future research.

As mentioned above, in a comprehensive meta-analysis, Gerber et al. [2] found no overall effect of social comparison on affect, instead highlighting the critical role of moderator variables, such as how comparison information is framed. Specifically, affective contrast effects were stronger when participants received manipulated feedback about both their own performance and that of a comparison standard. Our study employed a similar manipulation to induce threat. However, it diverges in one important aspect: Gerber et al. restricted their analysis to comparisons involving a single, specific individual, whereas our study utilized an aggregate comparison standard (i.e., feedback referencing performance relative to participants’ fellow students). Zell and Alicke [22] emphasize that local comparisons with specific individuals tend to dominate self-assessments when local and general (i.e., larger aggregates) feedback is presented simultaneously. They also note, however, that when general feedback is presented in isolation - as in our study - aggregate comparisons may still exert their effects. This highlights the importance of (identifying) boundary conditions.

On a cognitive level, a more threatening outcome resulted in increased rededication. Downplaying the importance of the task might have served a protective function for the high-threat group, suggesting a mechanism aimed at protecting self-esteem and alleviating the negative affect [23]. However, since we did not include an additional affect measure, the extent of this affective regulation remains speculative and warrants further research. Meanwhile, the observed lower commitment to future tasks in the high-threat group appears to be consistent with their higher levels of rededication, as both cognitive responses imply a lower willingness to allocate effort to the present task.

The present findings are also compatible with several other motivational and self-regulatory accounts and should therefore not be interpreted as uniquely diagnostic of gComp. These accounts would make partly similar predictions regarding rededication and reduced commitment after unfavorable feedback, but they emphasize different mechanisms and moderators. From a self-protection or self-esteem maintenance perspective, downplaying the importance of the task may protect self-worth by reducing the relevance of a domain in which one has ostensibly performed poorly [23]. Cognitive dissonance accounts would make a similar prediction if negative feedback conflicts with participants’ self-view as competent students: discounting the task’s relevance may reduce the inconsistency between “I am competent” and “I performed poorly” [24]. Self-discrepancy and regulatory focus accounts likewise suggest that unfavorable feedback may be especially aversive when it activates discrepancies between the actual self and salient ideal or ought standards [25], or when it is experienced in terms of failure prevention rather than growth and advancement [26]. Expectancy-value theory would predict reduced commitment when unfavorable feedback lowers either expectancy of future success or the subjective value of the task [27]. Finally, control-related and attributional accounts would predict lower commitment particularly when poor performance is attributed to stable or uncontrollable causes, but potentially greater renewed effort when the outcome is attributed to controllable or unstable causes [28, 29]. Thus, these perspectives converge with gComp in predicting that unfavorable evaluative feedback can elicit defensive devaluation or motivational disengagement, while also suggesting different moderators, such as self-relevance, perceived controllability, expectancy of improvement, and regulatory orientation.

Against this background, the specific contribution of gComp is not that it uniquely predicts rededication after unfavorable feedback. Rather, gComp provides an integrative framework for organizing affective, cognitive, and behavioral responses specifically in the context of comparative self-evaluation. In particular, the model links the valuation of a comparison outcome to distinct cognitive orientations, including rededication, distraction, reconstrual, and commitment, and further specifies how these orientations may be associated with behavioral assimilation or quiescence [6]. In this respect, gComp offers a more differentiated account of responses to threatening comparative feedback than frameworks that focus primarily on self-protection, dissonance reduction, expectancy, or control. In the following, we consider the extent to which gComp-derived predictions were supported.

Turning to the remaining cognitive orientations, scores of reconstrual and distraction did not differ significantly between the groups. gComp proposes that perceived high-threat may give rise to either rededication, distraction, or reconstrual, yet it does not delineate the conditions under which each pathway is expected to occur. In the present study, rededication appeared to be the predominant cognitive orientation. As an explanation, it may have been relatively easy for participants to downplay the significance of the task, given its context as a low-stakes online experiment rather than a high-stakes situation such as an academic exam or a job-related assessment. In higher-stakes scenarios, individuals may be more inclined to adopt distraction or reconstrual as coping strategies. Our finding that rededication, but not distraction or reconstrual, was associated with high-threat feedback suggests that certain upward social comparisons perceived as threatening may elicit only one, rather than all three, of the cognitive pathways proposed to follow a threatening social comparison outcome.

Future research should explore the conditions under which different cognitive orientation pathways emerge in response to threatening social comparisons. This includes examining contextual factors such as the relevance of the evaluated dimension and the characteristics of the comparison standard and the comparison context. Additionally, individual differences, such as self-concept, coping style, and regulatory focus [26], may influence the particular pathway individuals follow in response to threatening social comparisons. A more nuanced understanding of these moderating factors could clarify why certain individuals gravitate toward rededication, while others engage in distraction or reconstrual, thereby refining theoretical models of social comparison.

As mentioned above, gComp postulates that affective responses influence cognitive orientation, which, in turn, shape behavioral responses. In particular, when rededication or distraction occurs, behavioral quiescence is hypothesized to be a likely outcome. However, despite observed between-group differences in affect and rededication, no corresponding difference in behavior emerged. In fact, more than two-thirds of participants chose to repeat the task. One possibility for this finding is that rededication in the high-threat group was only partially achieved. Although participants in this group downplayed the task’s importance to a greater extent than those in the low-threat group, this rededication may not have been sufficiently strong to alleviate the discomfort associated with negative feedback. The decision to repeat the task may have served as a means of reducing dissonance-like discomfort [24], offering an opportunity to reaffirm their competence and obtain more favorable evidence about their performance. The low-cost (task duration < 1 min) and low-stakes nature of the task may have contributed to this outcome. That is, participants may have perceived minimal barriers to task repetition, resulting in a lack of significant behavioral differences between groups. Taken together, the present findings provide partial support for selected gComp assumptions: unfavorable performance feedback altered affective responses and was associated with greater rededication and lower commitment, but it did not produce significant differences in reconstrual, distraction, or immediate task repetition. Future research directly comparing gComp-derived predictions with alternative accounts discussed above is needed to establish the model’s distinct explanatory contributions.

The absence of an immediate behavioral effect may also relate to the temporal scope outlined in gComp. While the model makes assumptions about long-term behavior, the present study operationalized behavior in the short-term. It is, therefore, possible that behavioral differences emerge only over time, particularly following repeated exposure to salient upward comparisons. Such exposure has been linked to negative affect, reduced self-esteem, and increased mental health symptoms (e.g., 30), underscoring the need for further investigation. Accordingly, future research should pursue both the experimental manipulation of repeated exposure to upward comparisons and the measurement of longer-term behavioral outcomes. To address the latter, participants could be asked to re-engage in the task on a subsequent day, with the opportunity to practice in the interim. Variables such as practice time and frequency of page access could serve as meaningful behavioral outcomes.

A potential limitation of the present study lies in the assessment of cognitive orientation, which relied on single-item measures for each orientation, specifically developed for the present study. We acknowledge that single-item measures limit measurement precision. Their use was intended as part of an initial paradigm-development study. Consequently, evidence for their construct validity is currently limited. Furthermore, the items were phrased in a manner that primarily captured participants’ agreement with each type of cognitive response. It is therefore possible that items partly capture general tendencies and attitudes rather than the degree to which they *actually* engaged with each of the four theorized orientations, i.e., reconstrual, rededication, distraction or commitment, during task performance. For instance, the item assessing distraction read “*To what extent do you prefer to be doing something else other than the task right now?*”. An alternative phrasing, more directly targeting performance-related cognitions - such as “*To what extent did you find yourself thinking that you would prefer to be doing something entirely unrelated to your performance on the concentration task?*” - might have yielded different results. Future research should therefore develop and validate a comprehensive multi-item scale to more reliably assess cognitive orientation.

Another limitation is that perceived credibility of the performance feedback was not assessed directly. The feedback was designed to be difficult for participants to verify, as the main task did not provide trial-by-trial correctness feedback and participants were informed that their standing depended on both reaction time and error rate. Although only a minority of participants were classified as suspicious, suspicion did not differ by condition, and sensitivity analyses excluding suspicious participants yielded the same substantive pattern of findings, interrater agreement for the dichotomous suspicion code was modest. Moreover, the open-ended suspicion item provides only indirect evidence of feedback credibility. Relatedly, perceived threat, challenge, and coping capacity were not directly assessed, so the interpretation of the conditions as threat-like versus challenge-like remains inferential. Future studies should therefore include direct measures of credibility and threat-appraisal.

## Conclusion

The present findings provide initial and partial support for selected assumptions of gComp. Performance-related comparative feedback affected affective responses and some cognitive/motivational responses but did not affect immediate behavioral choice. Future studies should include direct appraisal measures and credibility checks, validated multi-item measures of cognitive orientation, and designs that isolate the comparative component of feedback. Further research is warranted to refine and validate assessment strategies addressing the model and elucidate the conditions under which its proposed mechanisms operate. Ultimately, this line of inquiry may inform the development of interventions aimed at promoting more adaptive forms of comparative thinking.

## Appendix A

In Tables A1-A5, we present the analyses, which also include data from participants with very low 2-back task performance (i.e., *Pr* values $\:\le\:$ 0; *N* = 143).

**Table A1:** Participants’ characteristics for each condition (*N* = 143)

	Low-threat (*n* = 70; 52 females, 18 males)	High-threat (*n* = 73; 54 females, 19 males)				
	M (SD)	M (SD)	t	df	*p*	d
Age	24.46 (3.43)	23.89 (4.01)	0.91	141	0.366	0.15
PHQ-9	3.33 (2.42)	3.27 (2.15)	0.14	141	0.887	0.02

Note. PHQ-9, Patient Health Questionnaire depression module

**Table A2:** Time × condition mixed anova for positive and negative affect (*N* = 143)

PANAS subscale	Condition	Time M (SD)		Condition × Time (df = 1, 141)		
		T1	T2	F	*p*	η²_*p*_
PA	Low-threat	29.6 (6.97)	30.6 (6.76)	17.94	< 0.001	0.113
	High-threat	29.0 (6.19)	25.9 (6.68)			
NA	Low-threat	15.3 (6.07)	15.2 (5.65)	14.30	< 0.001	0.092
	High-threat	13.4 (4.04)	16.2 (6.30)			

Note. PANAS, Positive and Negative Affect Schedule; PA = Positive Affect; NA = Negative Affect

**Table A3:** Cognitive responses for each condition (*N* = 143)

	Low-threat	High-threat				
	M (SD)	M (SD)	t	df	*p*	d
Rededication^a^	3.64 (1.60)	3.00 (1.70)	2.33	141	0.021^*^	0.39
Reconstrual	2.79 (1.52)	3.36 (1.81)	-2.05	138.74	0.043^*^	-0.34
Distraction	3.71 (1.61)	4.07 (1.96)	-1.18	141	0.240	-0.20
Commitment	3.97 (1.49)	2.27 (1.38)	7.07	141	< 0.001^**^	1.18

Note. Items were rated on a scale ranging from 1 (*not at all*) to 7 (*very much*)

^a^ Lower scores indicate higher levels of rededication

* *p* < .05, ** *p* < .001

**Table A4:** Number of participants repeating vs. not repeating the task by condition (*N* = 143)

	Low-threat	High-threat	Total
Repeated task	44	55	99
Did not repeat task	26	18	44
Total	70	73	

*Note*. χ ^2^ (1) = 2.62, *p* = .106

**Table A5:** 2-Back performance based on the bias-corrected discrimination index, Pr, for each condition* (N = 143)*

	Low-threat	High-threat				
	M (SD)	M (SD)	t	df	*p*	d
Trials 1–42	0.57 (0.38)	0.54 (0.29)	0.53	141	0.597	0.09
Trials 1–20	0.45 (0.40)	0.48 (0.32)	-0.52	141	0.602	-0.09
Trials 21–42	0.62 (0.37)	0.53 (0.30)	1.49	141	0.138	0.25

*Note. Pr = ((# hits + 0.5) / (# targets + 1)) – ((# false alarms + 0.5) / (# nontargets + 1)*

## Appendix B

**Table B1:** Cognitive response items and response options in German

	Item	Response options
Reconstrual	“Inwieweit haben gewisse Störfaktoren Ihre Leistung in der Konzentrationsaufgabe beeinflusst?”	1 = gar nicht, 7 = sehr stark
Rededication	“Für wie essentiell erachten Sie es, dass Sie bei der Konzentrationsaufgabe gut abgeschnitten haben?”	1 = gar nicht essentiell, 7 = sehr essentiell
Distraction	“Inwieweit verspüren Sie in diesem Moment den Wunsch, sich mit etwas anderem als der Konzentrationsaufgabe zu befassen?”	1 = gar nicht, 7 = sehr
Commitment	“Inwieweit ist Ihnen wichtig, dass Sie zukünftig Aufgaben wie die heutige besser absolvieren können?”	1 = gar nicht, 7 = sehr

## Data Availability

The anonymized data and analytical script are available on: https://osf.io/qkwrn/?view_only=3336c9f795c24f99821986ecaaae4009.

## Footnotes

1 We also developed and included an item to assess self-affirmation in this study. However, it will not be further examined here, as it pertains to a different research question.
